# In memoriam of prof. Francesco Pipino

**DOI:** 10.1007/s10195-014-0319-6

**Published:** 2014-09-28


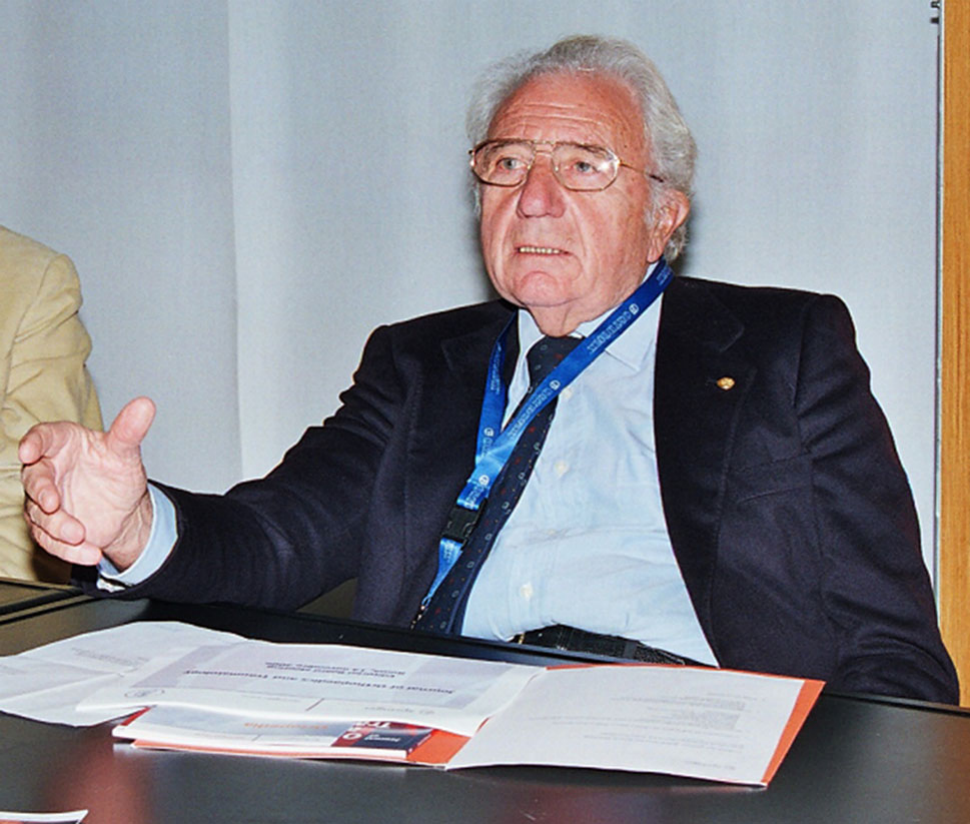


Francesco Pipino was a leading personality of Italian and international orthopedic community, a great scientist, a remarkable mentor, and a friend to us all.

Born in Ivrea in 1931, he studied Medicine and trained in orthopedic surgery at the University of Genoa in the late 1950s. By 1962 he was adjunct professor in the same university. To him, teaching was a mission and his career unfolded as Full Professor of Orthopedics and Director of the Residency program at the University of Bari from 1972 to 1993, and later at the University of Genoa from 1993 to 2003.

He was, at once, a prominent teacher and a fine surgeon––two roles he continued to fulfill at the University of Genoa where he became renowned for his impressive research activity. He pioneered the use of robotics in orthopedic surgery and was the creator of an innovative and revolutionary technique in hip replacement, the neck-preserving arthroplasty; he helped the concept of “less invasive” surgery to become a reality and gathered around him a school of talented surgeons.

Prof. Pipino founded six scientific societies, serving them as President, and was an honorary and an active member of prestigious international ones. In 2000, he founded the *Journal of Orthopaedics and Traumatology*, the official journal of *Italian Society of Orthopaedics and Traumatology.*

President of the *Italian Society of Orthopaedics and Traumatology* from 1996 to 1998, he is widely regarded as having been one of the pillars in the field of bone science both in Italy and in Europe. In public and in private he was always willing to discuss and debate, unfailingly courteous and firm in his convictions.

He will always remain in our hearts as an unforgettable teacher, a true friend and a perfect example of great surgeon and outstanding man.

Marco d’Imporzano

*Journal of Orthopaedics and Traumatology*

*Editor*-*in*-*Chief*

